# Incidence, risk factors and outcomes of acute kidney injury in surgical intensive care unit octogenarians at the Jordan University Hospital

**DOI:** 10.1186/s12877-023-03975-2

**Published:** 2023-05-04

**Authors:** Amjad Bani Hani, Mahmoud Abu Abeeleh, Sondos Al-Najjar, Abdulla Alzibdeh, Shahd Mansour, Isam Bsisu, Nour Awamleh, Randa Farah

**Affiliations:** 1 Department of General Surgery, School of Medicine, The University of Jordan, Amman, 11942 Jordan; 2 School of Medicine, The University of Jordan, Amman, 11942 Jordan; 3 King Hussein Cancer Center, Amman, 11942 Jordan; 4 Department of Anesthesia and Intensive Care, School of Medicine, The University of Jordan, Amman, 11942 Jordan; 5 Department of Internal Medicine, School of Medicine, The University of Jordan, Amman, 11942 Jordan

**Keywords:** Acute kidney injury, ICU, Octogenarians

## Abstract

**Background:**

Acute kidney damage (AKI) is among the most severe consequences observed in surgical intensive care units (SICUs). We aim to observe the incidence, risk factors, and outcomes of acute kidney injury in SICU octogenarians.

**Methods:**

A cross-sectional retrospective study was conducted at the SICU of Jordan University Hospital (JUH), a tertiary teaching hospital in a developing country, between January 2018 and December 2019. Patients who were 80 years or older at the time of data collection were included. The definition of AKI was based on Kidney Disease Improving Global Outcomes (KDIGO) criteria. Demographic, clinical, and laboratory data were reviewed.

**Results:**

A total number of 168 patients were included. The mean age was 84.0 ± 3.8 years, and 54.8% of the participants were women. Of those, 115 (68.5%) had surgery before or during ICU stay, and 28.7% of the patients’ surgeries were an emergency surgery. Also, 47.8% of surgeries were considered by anesthesia to be high-risk surgeries. A total of 55 patients (32.7%) developed AKI during their SICU stay. The factors that were significantly associated with AKI in the ICU patients included use of beta blocker [AOR: 3.7; 95% CI: 1.2–11.8; p = 0.025], and inotropes [AOR:4.0; 95% CI: 1.2–13.3; p = 0.03]. The factors that were significantly associated with mortality in the ICU included using mechanical ventilation [AOR:18.7; 95% CI: 2.4-141.9; p = 0.005] and inotropes use [AOR: 12.3; 95% CI: 1.2-120.7; p = 0.031].

**Conclusions:**

The incidence of AKI during SICU stay in this study was 32.7% and it was significantly associated with the use of beta blockers, mechanical ventilation, and inotropes. The mortality rate among octogenarians who developed AKI during SICU stay was 36.4%. Further studies are needed globally to assess the incidence of AKI in octogenarian surgical patients and identify risk factors to provide preventative measurements and strategies.

**Supplementary Information:**

The online version contains supplementary material available at 10.1186/s12877-023-03975-2.

## Background

Acute kidney injury (AKI) commonly complicates serious illnesses in hospitalized patients [1–5]. AKI can be identified by the evaluation of serum creatine (Cr) levels or urine output. The incidence of AKI in surgical intensive care unit (SICU) patients ranges from 16.7 to 30% [6–8]. The cause of AKI in SICU patients can be categorized into pre-renal, intrinsic, and post-renal. [6, 9, 10]

AKI is a significant condition itself that notably affects the morbidity and mortality rates of hospitalized patients. In addition to that, AKI increases costs and financial burden on hospitals, patients, and societies [11]. Accordingly, many studies investigated the risk factors associated with AKI in hospitalized patients generally, and in SICU patients specifically. Such studies found many factors that are related to the incidence of AKI in SICU patients, including patient factors such as age, sex, and comorbidities like diabetes mellitus and hypertension [6]. Others are related to the surgery, such as surgery time, type of operation, type of anesthesia, intraoperative complications, and postoperative management [4, 6, 12, 13].

The world’s population is getting older, with 11% of people aged over 60 [14]. This means that more people over 80, or octogenarians, constitute the patients to whom hospital services are provided. With ageing, functional, and anatomical changes occur to the kidneys which predisposes octogenarians to a bigger risk to develop renal problems. One such problem is AKI [15], especially among those who are hospitalized at the SICU units. In Jordan, the number of octogenarians is expected to rise from 41,000 (0.6% of population) to 254.000 (2.2% of population) in 2050 [16], which is a major demographic change that has implication on the patients’ population which the healthcare system needs to provide care for.

The amplitude of contributing risk factors is different between institutions. Identifying the major risk factors influencing the rate of AKI incidence in each institution would help in combating such factors and lowering the incidence of this condition, with the result of improving the outcomes and experiences of SICU patients. A study at King Abdullah II teaching and referral hospital in Jordan has identified several risk factors that increase the risk of AKI in hospitalized octogenarians [17], but did not study the incidence and risk factors in octogenarians at the SICU units. This study investigates the incidence, risk factors, and outcomes of AKI in SICU octogenarian patients at JUH.

## Materials and methods

This is a cross-sectional retrospective study that included all patients aged over 80 who were admitted to the SICU at the JUH, which is a referral tertiary teaching hospital located in Amman, the capital of Jordan, and provides medical services for patients from all parts of the country. We included all patients above the age of 80 who were admitted to the SICU between January 2018 and December 2019, whether they underwent surgical procedures or not, including cardiac surgical procedures. We excluded 20 patients who presented to the SICU with AKI, 6 patients who were end-stage renal disease on dialysis, and 15 patients with missing clinical data.

This study was approved by the Jordan Institutional Review Board, and all procedures performed were under the ethical standards of the institutional and/or national research committee, and with the principles of the World Medical Association Declaration of Helsinki.

Baseline characteristics at time of SICU admission were collected from hospital medical records by trained fifth- and sixth-year medical students, including demographic data, patients’ co-morbidities, medication used before ICU admission, and baseline vital signs at admission. Data about the cause of admission to the SICU was also identified. Baseline laboratory workup was collected as serum hemoglobin, white blood count, potential of hydrogen (pH), serum bicarbonate level (HCO_3_^-^), and serum albumin. Baseline measurements of renal function (measured by serum creatinine (SCr) and estimated glomerular filtration rate (eGFR) using CKD-EPI calculation were collected; CKD patients with a GFR of < 60 mL/min/1.75m^2^ were also included [18].

The Sequential Organ Failure Assessment (SOFA) scores were calculated for all patients in our cohort as a monitor of acute morbidity in critical care setting [19]. We identified type of surgery at SICU admission, and for patients who underwent surgical procedures we collected type of anesthesia as regional, spinal, and general anesthesia. Our institutional guidelines defined emergency surgeries as surgeries which were done within 72 h of admission.

During the SICU stay, we identified interventions such as respiratory support using non-invasive and invasive ventilation, hemodynamic support using vasopressors, blood transfusion, and type of antibiotics used. The primary outcome was the incidence of AKI in ICU patients. AKI was defined according to the Acute Kidney Injury Network (AKIN) criteria [20]. Serum creatinine was used as a criterion for diagnosing AKI, as urine output was not available for all patients. Hence, we followed AKIN criteria for the diagnosis of AKI: abrupt (within 48 h) reduction in kidney function, an absolute increase in SCr of 0.3 mg/dL or more (≥ 26.4 µmol/L), or a percentage increase in SCr of 50% or more (1.5-fold from baseline). For patients with CKD stage 3 and above, the 1.5-fold increase from baseline was used as a diagnostic criterion [21]. Major clinical outcomes were studied in relation with AKI, such as mortality, SICU stay in days, and days of hospital stay.

### Statistical analysis

All analyses were performed using STATA (Stata Statistical Software: Release 16. College Station, TX, StataCorp LLC). We presented categorical variables as percentages, and continuous variables are presented as the mean ± standard deviation.

The differences in sociodemographic characteristics, comorbidities, medications, vital signs, SOFA score, laboratory data, type of surgeries, SICU intervention and outcomes, and type of anesthesia among patients with or without AKI were assessed using the chi-square test for categorical variables and student’s t-test for continuous variables. Regression models were constructed to assess the variables associated with an increased risk of postoperative AKI. The potential baseline characteristics affecting AKI like age, comorbidities such as coronary artery disease and chronic kidney disease, medications such as **angiotensin-converting enzyme inhibitors (ACEi)**, proton pump inhibitors (PPI), metformin and betablockers, type of surgery and SOFA score were all assessed using univariable regression analysis, reporting the adjusted odds ratio (AOR) and the 95% confidence interval (95%CI). Additionally, the potential interventions during SICU stay affecting postoperative AKI (ventilator use, vasopressors use, blood transfusion, and antibiotics used such as vancomycin and fluroquinolones) and potential SICU outcomes which were affected by postoperative AKI, like hospital and SICU stay and mortality were evaluated. Only significant factors (P < 0.05) from the univariate analysis were fitted into the final multivariable regression model. The drop command was used to exclude variables with high variance inflation factor (VIF) to minimize multicollinearity. The confidence interval was set at 95% and p-values of ≤ 0.05 were considered to indicate statistical significance.

## Results

The characteristics of the population are shown in Table 1. The mean age of this cohort was 84.0 ± 3.8 years, ranging between 80 and 98 years, with 92 (54.8%) of the participants being females.

**Table 1:** The demographic data, past medical history, baseline investigations and laboratory data upon admission to SICU associated with AKI

Baseline characteristics	No AKI n = 113 (67.3%)	AKI n = 55 (32.7%)	Total Number n = 168	P value
**Age**				
Mean age (years ± SD)	83.7 ± 3.4	84.7 ± 4.6	84.0 ± 3.8	0.4
**Gender**				
Female	61(54.0%)	31 (56.4%)	92 (54.8%)	0.7
Male	52 (46.0%)	24 (43.6%)	76 (45.2%)	
Smoking	28 (24.8%)	15 (27.3%)	43 (27%)	0.7
BMI	29 ± 7	28.6 ± 5.4	28.9 ± 6.6	0.6
**Comorbidities**				
Hypertension	82 (72.6%)	45 (88.8%)	127 (75.5%)	0.2
Diabetes	46 (40.7%)	24 (43.6%)	70 (41.7%)	0.7
Coronary artery disease	42 (37.2%)	28 (50.9%)	70 (41.7%)	0.09
Chronic Pulmonary diseases	13 (11.5%)	3 (5.5%)	16 (9.5%)	0.2
Chronic renal diseases	38 (33.6%)	28 (50.9%)	66 (39.3%)	0.03 *
Neurological diseases	14 (12.4%)	11 (20.0%)	25 (14.9%)	0.2
Active malignancy	11 (9.7%)	3 (5.5%)	14 (8.3%)	0.35
**Medications**				
ACEI or ARB	32 (28.3%)	10 (18.2%)	42 (25%)	0.2
Aspirin	36 (31.9%)	23 (41.8%)	59 (35.1%)	0.2
Diuretics	24 (21.2%)	14 (25.5%)	38 (22.6%)	0.5
Statin	18 (15.9%)	15 (27.3%)	33 (19.6%)	0.08
Proton pump inhibitors	20 (17.7%)	13 (23.6%)	33 (19.6%)	0.3
Anticoagulation	29(25.7%)	19 (34.5%)	48 (28.6%)	0.2
Metformin	12 (10.6%)	1 (1.8%)	13 (7.7%)	0.05*
Beta blocker	27 (23.9%)	25 (45.5%)	52 (30.9%)	0.004*
**Vital signs**				
Mean arterial pressure	97.4 ± 16.7	87.0 ± 17.0	94.2 ± 17.0	< 0.001*
Respiratory rate	20.2 ± 3.8	20.1 ± 2.5	20.2 ± 3.4	0.5
Heart rate	87.5 ± 19.4	83.6 ± 20.6	86.2 ± 20	0.07
Sofa Score	3.2 ± 2.0	5.4 ± 2.7	3.9 ± 2.5	< 0.001*
**Laboratory Data**				
Hemoglobin level (g/dL)	11.5 ± 3.2	10.8 ± 2.2	11.35 ± 3.0	0.05*
White blood count(10^9^/L)	11.5 ± 6.7	11.9 ± 7.8	11.6 ± 7.1	0.8
pH	7.42 ± 0.07	7.40 ± 0.07	7.41 ± 0.7	0.08
Bicarbonate (meq/L)	22.9 ± 4.4	20.5 ± 5.65	22.0 ± 5.0	0.007*
eGFR (mL/min/1.73m^2^)	68.8 ± 19.4	60.6 ± 22.5	66.2 ± 20.7	0.02*
CRP (mg/dL)	117.6 ± 114.0	144.7 ± 134.2	127.1 ± 121.6	0.5
Albumin (g/dL)	3.3 ± 0.7	2.9 ± 0.7	3.2 ± 0.7	0.02*
**Type of surgery**				
Number of patients who underwent surgery	n = 83 (73.5)	n = 32 (58.2)	n = 115 (68.5)	0.046
Cardiovascular	9 (10.9%)	1 (3.1%)	10 (8.7%)	0.6
Gastrointestinal	38 (45.8%)	13 (40.6%)	51(44.3%)	
Neurology	2 (2.4%)	2 (6.3%)	4 (3.5%)	
Orthopedic	26 (31.3%)	13 (40.6%)	39 (33.9%)	
Plastic	4 (4.8%)	2 (6.3%)	6 (5.2%)	
Endocrinology	4 (4.8%)	1 (3.1%)	5 (4.3%)	
Emergency surgery	23 (27.7%)	10 (31.3%)	33 (28.7%)	0.7
High risk surgery	39 (47.0%)	16 (50.0%)	55 (47.8%)	0.7
**Type of anesthesia**				
Regional anesthesia	2 (2.4%)	3 (9.4%)	5 (4.4%)	0.2
Spinal anesthesia	18 (21.7%)	7 (21.9%)	25 (21.7%)	
General anesthesia	63 (75.9%)	22 (68.7%)	85(73.9%)	

AKI; Acute kidney injury, SICU; Surgical intensive care unit injury; e GFR; estimated glomerular filtration rate, PH; potential hydrogen, BMI; body mass index. ACEi; angiotensin converting enzyme inhibitors, ARBS; angiotensin receptor blockers. CRP; C-reactive protein

Of the total 168 patients who were included in this study, 70 (41.7%) had diabetes mellites (DM), 127 (75.5%) had Hypertension (HTN), 16 (9.5%) had pulmonary diseases, and 70 (41.7%) of patients had coronary artery diseases **[**Table 1**]**. The average estimated glomerular filtration rate (eGFR) at the time of admission was 66.2 ± 20.7 mL/min/1.73m^2^ and 66 (39.3%) of the patients had chronic kidney disease (CKD) (GFR < 60 mL/min/1.75m^2^). Moreover, 42 (25.0%) of patients were using angiotensin converting enzyme inhibitors (ACEi) / angiotensin receptor blockers(ARBs) as antihypertensive medications, 38 (22.6%) were using diuretics, 52 (30.9%) were using beta blockers, and 13 (7.7%) of patients used metformin before admission.

In our study population, 115 (68.5%) had surgery before or during ICU stay. The most common surgery was gastrointestinal (44.3%), followed by orthopedic surgery (33.9%) and cardiovascular surgery (8.7%). In the studied population, 33 (28.7%) of the performed surgeries were emergency surgeries, while 55 (47.8%) of surgeries were considered by anesthesia as high-risk surgery. Eighty-five (73.9%) of the patients received general anesthesia while 25 (21.7%) spinal anesthesia.

A total of 55 patients (32.7%) developed AKI during their SICU stay. The demographic data, type of surgery before ICU admission, comorbidities, regular home medications, vital signs at the time of admission, in addition to basic laboratory data in admission to ICU intervention, and type of surgery are presented in **[**Table 1**]**.

### Acute kidney injury incidence and risk factors

Of our patient population, 32.7% (55) developed AKI. The univariate analysis of baseline characteristics and their association with AKI during SICU stay was done and the results indicate that patients with chronic kidney disease showed a statistically significant association with AKI with (p = 0.031), with 28 (50.9%) of patients who developed AKI during their SICU stay were previously diagnosed with chronic kidney diseases. Moreover, patients who were taking betablockers had a higher risk of developing AKI (p = 0.004) and patients who were taking metformin are at lower risk of developing AKI with a (p = 0.0046) **[**Table 1**]**. The analysis also showed that lower mean arterial pressure at time of admission to the ICU was significantly associated with higher risk of AKI compared to higher readings (p < 0.001). In addition, lower bicarbonate levels were significantly associated with AKI (p = 0.007), and lower baseline GFR is significantly associated with higher risk of AKI (p = 0.021). Additionally, higher SOFA score at time of admission was significantly associated with higher risk of AKI in SICU patients with significant (p < 0.001) **[**Table 1**]**.

### Acute kidney injury relations with interventions and outcome

The results of the univariate analysis of SICU intervention and outcomes and their association with AKI indicate that use of fluoroquinolones showed a statistically significant association (p = 0.04), but no significant association was found between the use of aminoglycoside and vancomycin with AKI (p = 0.8). Moreover, we found that use of ventilators, inotropes and blood transfusion during ICU stay were significantly associated with higher risk of AKI (p = 0.004, < 0.001 and 0.04 respectively). AKI in ICU was not found to have a significant impact on ICU or hospital stay length with (p = 0.2 and 0.09 respectively), with an average SICU stay of 6 ± 8 days and average hospital stay of 12.9 ± 11.1 days for those who developed AKI. Mortality had a significant association with AKI (p < 0.001), with a mortality rate of 19% among those who developed AKI, compared to 10.6% among those who did not. **[**Table 2**]**.

**Table 2:** SICU Interventions and Outcomes associated with AKI.

Characteristic	No AKI n = 113 (67.3%)	AKI n = 55 (32.7%)	Total Number n = 168	P value
**Interventions**				
Ventilators	10 (8.8%)	14 (25.5%)	24 (14.3%)	0.004*
CPAP use	14 (12.4%)	10 (18.2%)	24 (14.3%)	0.31
Inotropes	15 (13.3%)	30 (54.5%)	45 (26.8%)	< 0.001*
Blood Transfusion	13 (11.5%)	13 (23.6%)	26 (15.5%)	0.04*
**Antibiotics**				
Aminoglycosides	5 (4.4%)	2 (3.6%)	7(4.2%)	0.8
Vancomycin	47 (41.6%)	24 (43.6%)	71 (42.2%)	0.8
Fluoroquinolones	30 (26.5%)	23 (41.8%)	53 (31.5%)	0.04*
**Clinical outcomes**				
Hospital stays (days)	9.8 ± 8.1	12.9 ± 11.1	10.8 ± 9.3	0.09
SICU stay (days)	4.2 ± 4.8	6.0 ± 8.0	4.8 ± 6.0	0.2
Mortality	12 (10.6%)	20 (36.4%)	32 (19.0%)	< 0.001*

AKI = Acute kidney injury, SICU = Intensive care unit injury; CPAP: continuous positive airway pressure

## Mortality incidence and risk factors

During SICU stay, **19.9% (32)** of the patients died. The univariate analysis of baseline characteristics and their association with mortality during SICU stay was done and the results indicate that patients with diabetes and coronary artery disease had a statistically significant association with mortality with (p = 0.05, and 0.02) respectively. **[Supplemental Table 1]**. The data also showed that patients with lower mean arterial pressure at time of admission to the SICU had a significant higher risk of mortality compared to higher readings (p = 0.04). In addition, lower serum albumin levels were significantly associated with mortality (p = 0.003), and higher c reactive protein level was significantly associated with higher risk of mortality (p = 0.04). Additionally, higher sofa score at time of admission was significantly associated with higher risk of mortality in SICU patients with significant (p < 0.001) **[Supplemental Table 1]**.

### Mortality relations with interventions and outcome

The results of the univariate analysis of SICU intervention and outcomes and their association with mortality indicate that use of fluoroquinolones showed a statistically significant association (p = 0.02), but no significant association was found between the use of aminoglycoside and vancomycin with AKI (p = 0.3, 0.8) respectively. Moreover, we found that use of ventilators, inotropes and blood transfusion during ICU stay were significantly associated with higher risk of mortality (p < 0.001, < 0.001 and 0.002 respectively). Mortality in SICU was associated with longer SICU stay with a p value = 0.01 but with not associated with longer hospital stay length with (p = 0.9). AKI had a significant association with mortality (p < 0.001) **[Supplemental Table 2 ]**.

### Multivariate Regression

A binary logistic regression model was used to analyze significant variables from the univariate analysis (comorbidities, medications, vital signs, at admission, AKI during ICU stay and laboratory results, SOFA score, ICU interventions and antibiotics use during ICU stay). After adjusting for known confounders, the factors that were significantly associated with AKI in the ICU patients included history of beta blocker use (AOR: 3.7; 95% CI: 1.2–11.8; p = 0.025), and use of inotropes (AOR:4.0; 95% CI: 1.2–13.3; p = 0.03). However, SOFA score was not associated with AKI (AOR:1.3; 95% CI: 0.99–1.8; p = 0.06) **[**Table 3**]**.

**Table 3:** Binary logistic regression analysis of factors associated with SICU AKI.

Variables	OR (95% CI)	p-value
**Baseline characteristics**		
Mean Arterial Blood Pressure	1.0 (0.96–1.03)	0.81
Serum albumin	0.7 (0.33–1.5)	0.37
Bicarbonate	0.9 (0.86–1.1)	0.3
Hemoglobin	1.1 (0.8–1.5)	0.4
eGFR	1.0 (0.96–1.01)	0.5
**SOFA Score**	1.3 (0.99–1.8)	**0.06**
**Medications at admission**		
Beta blocker use	3.7(1.17–11.8)	0.025*
Metformin	0.33 (0.015-7.4)	0.5
**Interventions during SICU stay**		
Ventilators	0.9 (0.22–4.3)	0.8
Inotropes	4.0 (1.2–13.3)	0.03*
Blood transfusion	2.1 (0.5–8.6)	0.3
Fluoroquinolones	0.7 (0.23–2.1)	0.5

Included in the multiple logistic regression analysis; AKI = Acute kidney injury, SOFA = sequential Organ Failure Assessment, SICU = Surgical intensive care unit injury; e GFR = estimated glomerular filtration rate CI = confidence interval, OR = Odds ratio.

Use of ventilators during ICU stay (AOR:18.7; 95% CI: 2.4-141.9; p = 0.005) and use of inotropes were significantly associated with higher risk of mortality in surgical ICU (AOR: 12.3; 95% CI: 1.2-120.7; p = 0.031). We were not able to identify a significantly increased risk of mortality in patients who developed AKI during their ICU stay (AOR: 1.5; 95% CI: 0.43–5.27; p = 0.2). Moreover, we did not appreciate the relation between SOFA score and mortality (AOR: 1.2; 95% CI: 0.7–1.8; p = 0.6) **[**Table 4**]**.

**Table 4:** Binary logistic regression analysis of factors associated with SICU Mortality

Variables	OR (95% CI)	p-value
**Baseline characteristics**		
Mean arterial blood pressure	1.0 (0.91–1.03)	0.2
SOFA Score	1.2 (0.7–1.8)	0.6
Serum Albumin	0.5 (0.13-2.0)	0.3
C-reactive protein	1.0 (0.99–1.01)	0.2
Diabetes mellites	2.2 (0.35–14.3)	0.4
Coronary artery disease	1.4 (0.23–9.8)	0.7
**Interventions during SICU stay**		
Ventilators	18.7 (2.4-141.9)	0.005*
Inotropes	12.3 (1.2-120.7)	0.031*
Blood transfusion	1.4 (0.2–12.4)	0.8
Fluoroquinolones	0.4 (0.06–2.2)	0.3
SICU complications		
SICU stay	1.1 (0.98–1.2)	0.1
AKI	1.5 (0.43–5.27)	0.2

Included in the multiple logistic regression analysis; SOFA = sequential Organ Failure Assessment, AKI = Acute kidney injury, SICU = Surgical intensive care unit injury; CI = confidence interval; OR = Odds ratio.

## Discussion

Acute kidney injury (AKI) is a cause of significant morbidity in hospitalized patients including octogenarians [22]. In this study, the incidence of AKI was 32.7%, compared to many other studies where the rate varies between 20% [23] and 40% [24] of ICU patients above 80 years old. Between all AKI-related hospitalizations, 30–40% happen postoperatively [23]. AKI is an underdiagnosed problem but is considered a significant cause of morbidity and mortality postoperatively in both cardiac and non-cardiac surgeries, since it is associated with life-threatening complications such as sepsis, coagulopathy, and increased need for mechanical ventilation. [25–30].

The population of this study is octogenarians, and advanced age was widely described as an independent risk factor for the development of AKI [2, 29] and a risk factor for AKI in perioperative settings in cardiac and non-cardiac surgeries [2, 9, 31]. However, in our univariate analysis, no significant difference was found between the patients in the AKI group and non-AKI group in age (p = 0.4, Table 1). As explained by [32], octogenarians could be used to their chronic-reduced renal function and they might not be suffering so often from a worse renal function postoperative and without causing any relevant clinical consequences.

Postoperative acute kidney injury (PO-AKI) is defined as an AKI developing within seven days of an operation, according to the KDIGO definition of AKI [33]. We do recommend future studies dedicated towards investigating PO-AKI thoroughly in geriatric patients, since PO-AKI is a major complication and is associated with higher postoperative morbidity and mortality. These studies require a different study design and need to include all patients developing PO-AKI, not just those admitted to the ICU, in order to obtain all relevant data that can help in identifying perioperative risk factors for AKI. These factors include intraoperative blood loss, type and amount of colloids and crystalloids used, anesthetic medications used, number of episodes of hypotension and their duration, intraoperative position, and the duration of surgery [34].

We assessed several chronic comorbidities including hypertension, diabetes, coronary artery disease, chronic pulmonary diseases, chronic renal disease (with GFR of less than 60), chronic liver disease and active malignancy (Table 1). Chronic renal disease was the only significant commorbidity in our study and 50.9% of the AKI group had chronic renal dysfunction, in comparison to 33.6% in the non-AKI group (P = 0.031). This is seen in other studies as well, and chronic kidney disease stage III, IV, and V are are cited as independent risk factors for postoperative AKI in octogenarians [35, 36]. This could be due to fibrosis in CKD patients’ kidneys, as they become unable to respond to acute hemodynamic changes and prone to relative hypoxia [37]. Additionally, significantly higher serum HCO_3_^−^ and GFR levels were noticed in the non-AKI group, in comparison to the AKI group. It is thought that sodium bicarbonate contributes to increasing oxygen delivery to the renal medulla, while reducing iron-mediated free radical formation due to neutralizing acidosis in this vulnerable region of the kidney [38].

Of the medications that were analysed, only metformin and beta blockers were significantly related to the occurance of AKI (P value = 0.05). Metformin had a protective effect on patients in our cohort, since 10.7% of patients in the non-AKI group used metformin, compared to only 1.9% being on metformin in the AKI group. On the other hand, only beta blockers showed significant correlation with AKI in the bivariate and multiple logestic regression model of analysis (p = 0.025), with an OR of 3.7. Beta blockers were described in many studies as an independent protective factor for AKI, but we found a contradictory result. This can be explained by the difference in our age groups, as none of the studies were specifically addressing octogenarians. Beta blockers are poorly tolerated in the elderly, likely due to their inherent unfavorable effect on systemic hemodynamics and pathophysiologic findings in the arterial tree, heart, kidneys, and brain [39]. The reduction in arterial blood pressure and cardiac output may increase the risk of renal hypoperfusion and AKI in elderly patients [40]. Although it has been demonstrated that there is non-significant lower renal artery blood flow in patients receiving beta-blockers in the study of Hall et al. [41], these changes might be less tolerated by critically ill octogenarians. Nonetheless, 41.7% of patients in our study had history of coronary artery disease, and beta blockers reduce mortality in these patients [42]. Moreover, beta blockers can ameliorate renal function by reducing sympathetic activation and renal oxygen consumption [41, 43]. Therefore, although beta blockers reduced mortality in critical ill patients [44], we recommend future randomized controlled trials on octogenarians to determine the optimal target group of patients, choice of beta blocker, timing of treatment, and the optimal hemodynamic targets.

Hypotension during surgery has been associated with a higher incidence of AKI [45–48]. It is recommended to keep the mean arterial pressure (MAP) higher than 60–65 mm Hg, or higher than 75 mm Hg in hypertensive patients, in the ICU [49, 50]. In our sample, a statistically significant difference was found in mean arterial pressure between the two groups; AKI group had paradoxically higher MAP than non-AKI group, which contradicts what was described in previous studies. This could be due to their baseline co-morbidities and smoking status.

Our results also show that SOFA score is a strong predictive tool for AKI in geriatric patients in SICU. These findings were consistent with the previous studies [51–54]. Additionally, in Hai Wang et al. research in 2020, SOFA score showed a higher accuracy of mortality prediction in critically ill patients with AKI undergoing continuous renal replacement therapy than other scores. [55].

The incidence of AKI was found to be significantly higher in patients who needed mechanical ventilation (25.5% of the AKI group), when compared with those who did not need it (8.8% of the non-AKI group). This is consistent with what we can find in the literature; the state of dependence on ventilator was found to be associated with an increased incidence of AKI [29]. Mechanical ventilation requiring high airway pressure to maintain gas exchange reduces renal perfusion, due to higher hydrostatic pressure over the entire venous compartment [56]. Evidence suggests that a fall in atrial transmural pressure caused by positive end-expiratoy pressure (PEEP) in mechanical ventilation activates the sympathetic pathway, vasoconstricting afferent renal arteriole directly or through the activation of renin, resulting in a fall in renal perfusion and a faulty autoregulatory mechanism, causing a decline in GFR. Decreased blood flow reduce the delivery of sodium to the distal tubule, with renin/angiotension/ aldosterone activation and more sodium avidity, clincially leading to a fall in urine output. This is often treated with saline, diuretics, or dopamine, which all override the kidney’s compensatory mechanisms resulting in adverse consequences like fluid overload, intravascular volume depletion, and prerenal failure. [57].

Likewise, the use of inotropic agents was related to the incidence of AKI; 54.5% of those who developed AKI needed the use of inotropic agents during their ICU stay (versus 13.3% of those who did not develop AKI). As demonstrated by [58], patients who developed AKI needed more inotrope therapy despite receiving more infused fluid, indicating that the fluid therapy was insufficient. This is due to capillary leak syndrome resulting in intravascular hypovolemia and hemodynamic instability. Early and aggressive fluid therapy and dobutamine for cardiac output can possibly restore the balance between renal oxygen supply and demand, and AKI development.

In conclusion, the incidence of AKI during SICU stay in this study was 32.7% and it was significantly associated with the use of beta blockers, mechanical ventilation, and inotropes. AKI affected the morbidity and mortality rates of hospitalized octogenarian patients. The average SICU stay was 6 ± 8 days and average hospital stay was 12.9 ± 11.1 days for those who developed AKI. The mortality rate among octogenarians who developed AKI during SICU stay was 36.4%. Further studies are needed globally to assess the incidence of AKI in octogenarian surgical patients and identify risk factors to provide preventative measurements and strategies.

## Electronic supplementary material

Below is the link to the electronic supplementary material.

**Supplemental Table 1.** The demographic data, past medical history, baseline investigations and laboratory data upon admission to SICU associated with mortality. **Supplemental Table 2.** SICU Interventions and Outcomes associated with mortality.

## Data Availability

The datasets used and/or analysed during the current study are available from the corresponding author on reasonable request.
