# Supplementary material for: Incidence, risk factors and outcomes of acute kidney injury in surgical intensive care unit octogenarians at the Jordan University Hospital
Source: BMC Geriatr. 2023 May 4;23:266. doi: 10.1186/s12877-023-03975-2 (PMC10158325; doi:10.1186/s12877-023-03975-2)
Supplement: Supplementary file 1 — Supplemental Table 1. The demographic data, past medical history, baseline investigations and laboratory data upon admission to SICU associated with mortality. Supplemental Table 2. SICU Interventions and Outcomes associated with mortality. [file 12877_2023_3975_MOESM1_ESM.docx]

**Supplemental Table 1:** The demographic data, past medical history, baseline investigations and laboratory data upon admission to SICU associated with mortality.

| **Baseline characteristics** | **No death** | **Death** | **Total Number** | **P value** |
| --- | --- | --- | --- | --- |
|  | **n= 129 (80.1%)** | **n=** **32 (19.9%)** | **n= 161** |  |
| **Age** | | | | |
| Mean age (years ± SD) | 83.8 ± 3.5 | 85.3 ± 5.0 | 84.1 ± 3.9 | 0.2 |
| **Gender** | | | | |
| Female | 67 (51.9%) | 18 (56.2%) | 85 (52.8%) | 0.7 |
| Male | 62 (48.0%) | 14 (43.8%) | 76 (47.2%) |  |
|  | | | | |
| Smoking | 32 (24.8%) | 10 (31.3%) | 42 (26.1%) | 0.3 |
| BMI | 29 ± 7 | 26.4 ± 4.2 | 28.5 ± 6.4 | 0.1 |
| **Comorbidities** | | | | |
| Hypertension | 96 (74.4%) | 26 (81.3 %) | 122 (75.8%) | 0.4 |
| Diabetes | 47 (36.4%) | 18 (56.3%) | 65 (40.4%) | **0.04*** |
| Coronary artery disease | 47 (36.4%) | 19 (59.4%) | 66 (41.0%) | **0.02*** |
| Chronic Pulmonary diseases | 11 (8.5%) | 5 (15.6%) | 16 (10.0%) | 0.2 |
| Chronic renal diseases | 51 (39.5 %) | 14 (43.8) | 65 (40.4%) | 0.6 |
| Neurological diseases | 15 (11.6%) | 7 (21.9%) | 22 (13.7%) | 0.1 |
| Active malignancy | 12 (9.3%) | 0 (0%) | 12 (7.5%) | 0.07 |
| **Medications** | |  |  |  |
| ACEI or ARB | 31 (24.0 %) | 7 (21.9 %) | 38 (23.6 %) | 0.8 |
| Aspirin | 46 (35.7 %) | 10 (31.3%) | 56 (34.8%) | 0.7 |
| Diuretics | 30 (23.3 %) | 4 (12.5%) | 34 (21.1 %) | 0.2 |
| Statin | 26 (20.2%) | 7 (21.9 %) | 33 (20.5%) | 0.8 |
| Proton pump inhibitors | 25 (19.4 %) | 6 (18.8 %) | 31 (19.3%) | 0.9 |
| Anticoagulation | 35 (27.1%) | 12 (37.5%) | 47 (29.2%) | 0.2 |
| Metformin | 11 (8.5%) | 1 (3.1 %) | 12 (7.5%) | 0.3 |
| Beta blocker | 40 (31.0 %) | 11 (34.3 %) | 51 (31.7%) | 0.7 |
| **Vital signs** | | | | |
| Mean arterial pressure | 95.4 ± 17.1 | 89.2 ±17.0 | 94.1 ± 17.2 | **0.04*** |
| Respiratory rate | 20.3 ± 3.8 | 20.0 ± 2.3 | 20.2 ± 3.5 | 0.9 |
| Heart rate | 86.3± 20.4 | 85.2 ± 18.8 | 86.1 ± 20 | 0.7 |
| Sofa Score | 3.3± 2.0 | 6.0 ± 2.7 | 3.9 ± 2.5 | **<0.001*** |
| **Laboratory Data** | | | | |
| Hemoglobin level (g/dL) | 11.5 ± 3.2 | 11.0 ± 2.1 | 11.4 ± 3.0 | 0.5 |
| White blood count(10^9^/L) | 11.7 ± 7.3 | 11.6 ± 6.7 | 11.6 ± 7.1 | 0.8 |
| pH | 7.43 ± 0.07 | 7.40 ± 0.09 | 7.41 ± 0.07 | 0.1 |
| Bicarbonate (meq/L) | 22.3 ± 4.5 | 20.8 ± 6.1 | 22.0 ± 5.0 | 0.2 |
| eGFR (mL/min/1.73m^2^) | 66.8 ± 19.8 | 61.3 ± 25.1 | 65.7 ± 20.9 | 0.3 |
| CRP (mg/dL) | 176.7 ± 148.3 | 112.9 ± 110.3 | 127.7 ± 122.5 | **0.04*** |
| Albumin (g/dL) | 3.3 ± 0.7 | 2.8 ± 0.8 | 3.2 ± 0.7 | **0.003*** |
| **Type of surgery** | | | | |
| Number of patients who underwent surgery | n= 98 (76.0%) | n= 17 (53.1%) | n= 114 (70.8) |  |
| Cardiovascular | 7 (7.1%) | 3 (17.6 %) | 10 (8.8%) | 0.07 |
| Gastrointestinal | 47 (48.0%) | 4 (23.5%) | 51 (44.7 %) |  |
| Neurology | 2 (2.0%) | 2 (11.7%) | 4 (3.5%) |  |
| Orthopedic | 32 (32.7%) | 6 (35.3%) | 38 (33.3%) |  |
| Plastic | 5 (5.1%) | 2 (11.8%) | 6 (5.3%) |  |
| Endocrinology | 5 (5.1%) | 0 (0%) | 5 (4.4%) |  |
| Emergency surgery | 29 (29.6%) | 4 (23.5%) | 33 (29.0%) | 0.6 |
| High risk surgery | 48 (49.0%) | 7 (41.2%) | 55 (48.2 %) | 0.5 |
| Type of anesthesia | | | | |
| Regional anesthesia | 5 (5.1 %) | 0 (0.0%) | 5 (4.4%) | 0.5 |
| Spinal anesthesia | 19 (19.4 %) | 5 (29.4 %) | 24 (21.1%) |  |
| General anesthesia | 73 (74.5%) | 12 (70.6%) | 85 (74.6%) |  |
| AKI; Acute kidney injury, SICU; Surgical intensive care unit injury; e GFR; estimated glomerular filtration rate, PH; potential hydrogen, BMI; body mass index. ACEi; angiotensin converting enzyme inhibitors, ARBS; angiotensin receptor blockers. CRP; C-reactive protein | | | | |

**Supplemental Table 2:**  SICU Interventions and Outcomes associated with mortality.

| **Characteristic** | **No mortality**  **n= 129 (80.1%)** | **Mortality**  **n= 32 (19.9%)** | **Total Number**  **n= 161** | **P value** |
| --- | --- | --- | --- | --- |
| **Interventions** | | | | |
| Ventilators | 7 (5.4%) | 16 (50.0 %) | 23 (14.3%) | **<0.001*** |
| CPAP use | 15 (11.6%) | 6 (18.8 %) | 21 (13.0%) | 0.3 |
| Inotropes | 17 (13.2%) | 24 (75%) | 41 (25.5%) | **<0.001*** |
| Blood Transfusion | 15 (11.6%) | 11 (34.4%) | 26 (16.1 %) | **0.002*** |
| **Antibiotics** | | | | |
| Aminoglycosides | 4 (3.1%) | 3 (9.4%) | 7(4.3%) | 0.3 |
| Vancomycin | 55 (42.6%) | 14 (43.8%) | 69 (42.9%) | 0.9 |
| Fluoroquinolones | 33 (25.6%) | 15 (46.9%) | 48 (29.8%) | **0.02*** |
| **Clinical outcomes** | | | | |
| Hospital stays (days) | 10.2 ± 8.0 | 12.6 ± 12.9 | 10.7 ± 9.2 | 0.8 |
| SICU stay (days) | 4.3 ± 5.7 | 7.2 ± 7.5 | 4.9 ± 6.2 | **0.01*** |
| AKI | 32 (24.8%) | 20 (62.5%) | 52 (32.3%) | **<0.001*** |
| AKI= Acute kidney injury, SICU = Intensive care unit injury; CPAP: continuous positive airway pressure | | | | |
